# Exploration of Choroidal Thinning Located Temporal to the Fovea: A Pilot Study

**DOI:** 10.3390/jcm13174978

**Published:** 2024-08-23

**Authors:** Adèle Ehongo, Georgina Jawdat De Togme, Viviane De Maertelaer

**Affiliations:** 1 Hôpital Universitaire de Bruxelles (HUB), CUB Hôpital Erasme, Service d’Ophtalmologie, Route de Lennik 808, 1070 Bruxelles, Belgium; georgina.jawdat.de.togme@ulb.be; 2 Biostatistics, IRIBHM Université Libre de Bruxelles (ULB), Route de Lennik 808, 1070 Bruxelles, Belgium; viviane.de.maertelaer@ulb.be

**Keywords:** myopia, posterior staphyloma, choroidal thinning, pathogenesis, complication, muscle

## Abstract

**Background/Objectives:** Posterior staphyloma (PS) is a hallmark of pathological myopia, corresponding to a circumscribed outpouching of the eyeball with choroidal thinning and inward scleral deformation at its edges. Its pathogenesis is still unclear, thus constituting a research priority as the prevalence of myopia is increasing worldwide. Recently, it has been suggested that the optic nerve sheaths or oblique muscles are potential promoters of PS through the traction or compression effect that they apply to the eye wall. The inferior oblique muscle (IOM) inserts 1–2 mm from the macula. The projection of its insertion is accessible using Optical Coherence Tomography (OCT). Before launching prospective studies, we sought to detect any choroidal thinning (ChT) in the temporal vicinity of the macula and to measure the distance between it and the fovea (FT-distance). **Methods**: This retrospective cross-sectional pilot study included 120 eyes. Using Spectralis^®^-OCT, the area centered by the Bruch’s membrane opening–fovea axis was analyzed for ChT and FT-distance. **Results**: Of the 112 defined eyes, 70% (78 eyes) had ChT. Pachymetry was significantly thinner (*p* = 0.018) in eyes with than without ChT. The mean FT-distance was 3601.9 ± 93.6 µm. **Conclusions:** The location of ChT coincided with the insertion distance of the IOM, suggesting a link between them. The association between the presence of ChT and a thinner pachymetry suggests a reduced scleral resistance, as a thinner pachymetry is related to a thinner sclera. Our results suggest a link between ocular deformation and the IOM, which may be relevant for the pathogenesis of PS, warranting further investigation.

## 1. Introduction

The strategies currently mobilized against myopia aim to reduce its increasing prevalence as well as the vision-threatening complications linked to this condition. It is estimated that 50% of people worldwide will be myopic by 2050, compared to 23% in 2016 [1]. These figures could be revised upwards due to the influence of COVID-19 [2].

Although posterior staphyloma (PS) is one of the main myopic complications leading to poor visual prognosis [3], its pathogenesis is still poorly understood.

Interestingly, it has recently been suggested that peripapillary staphyloma (PPS) results from the long-term remodeling and fixation of intermittent eye wall deformations induced by the optic nerve (ON) sheaths on their scleral attachments during eye movements [4,5].

Indeed, the traction of the ON sheaths on their scleral insertions during eye movements has been systematically documented by various methods [6,7,8,9,10,11]. Moreover, the magnitude of this pulling force reaches that of the extraocular muscles [12].

Therefore, it was hypothesized that oblique muscles which also insert at the back of the globe could potentially favor the appearance of other types of PS, through the same mechanism of sectoral compression and/or traction of the eyeball [13].

As the insertion of the inferior oblique muscle (IOM) is located 1–2 mm from the macula [14], the potential deformations of the eyeball induced by it could be practically evaluated using Optical Coherence Tomography (OCT).

The aim of this work was therefore to look for localized choroidal thinning (ChT) in the temporal vicinity of the macula, using the serial scans that constituted the acquisition of the posterior pole of Spectralis^®^ OCT. In addition, this study aimed to measure the distance between this potential thinning and the fovea (FT-distance).

## 2. Materials and Methods

This cross-sectional study was conducted in the glaucoma outpatient department. It complies with the tenets of the Declaration of Helsinki and was approved by the Ethics Committee (reference P2023/423) and the institutional review board (reference SRB2023264).

The records of adult patients who underwent SD-OCT imaging with Spectralis^®^ S3300 model, version 6.16.2 (Heidelberg Engineering GmbH, Heidelberg, Germany) for glaucoma evaluation were retrospectively analyzed. The inclusion was carried out consecutively until 120 eyes were obtained.

As the acquisition of the posterior pole with the Spectralis^®^ Glaucoma Premium Edition module does not require dilation, tropicamide–phenylephrine mydriatic eye drops, which have been shown in a recent study to induce temporal choroidal thinning, were not used [15].

Due to the retrospective design, the informed consent was waived. However, for each medical file, the “clinical research” section was checked beforehand, and if the patient had formally objected to the use of their data for study purposes, their wish was respected, and they were not included.

Inclusion criteria: a quality score of the OCT scan of at least 25, visible and sharp posterior choroidal wall.

Exclusion criteria: An OCT signal quality below 25, non-myopic maculopathy, non-glaucomatous neuropathy, a history of trauma, a history of glaucoma surgery, a history of posterior segment surgery or strabismus surgery. An absence of visibility of the posterior choroidal wall, or a lack of posterior choroidal wall sharpness.

### 2.1. Data Acquisition

#### 2.1.1. General Parameters

The age and gender of the patients were recorded. For each eye, the refractive error (RE) in spherical equivalent, visual acuity (LogMar), central corneal thickness (CCT) with Pentacam^®^ (OCULUS Optikgeräte GmbH, Wetzlar, Germany, version 1.22r03), and the axial length (AL) if present, were measured with the IOL master^®^ 700 (Carl Zeiss Meditec AG, Jena, Germany Software version 1.70.16.55256), and were recorded. The RE of eyes with a history of refractive surgery or phacoemulsification was not considered, as well as the CCT of eyes with a history of refractive surgery.

#### 2.1.2. OCT Analysis

The OCT and infrared (IR) images were opened in display mode and the following analyses were performed.

- A search for choroidal thinning in 3 locations in the temporal part of the posterior pole.

**Figure 1:**
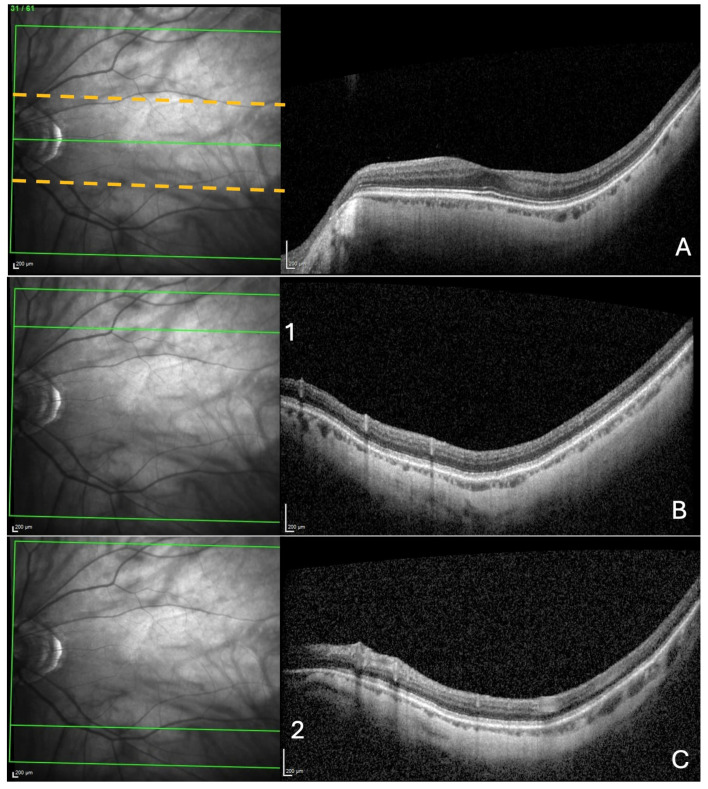
Search for choroidal thinning in 3 locations. (**A**) In a band (delineated by the dashed yellow lines), including 10 cross-sections on either side of the fovea–Bruch’s membrane opening axis. (**B**) In the upper limit of acquisition rectangle (10 cross-sections) delineated by the green line (1) in the IR image. (**C**) In the inferior limit of acquisition rectangle (10 cross-sections) delineated by the green line (2) in the IR image.

This was performed, respectively, near the fovea, and at the upper and lower limits of the acquisition rectangle. First, the rectangle acquisition band centered by the Bruch’s membrane opening–fovea (Fo-BMO) axis and including 10 sections below and 10 sections above this axis was assessed for the presence of a ChT (Figure 1A).

Then, from the upper limit of the rectangle acquisition, ten outer sections were analyzed, looking for choroidal thinning in the same manner as described for the ChT (Figure 1B). Finally, the same procedure was performed in the lower limit of the acquisition rectangle (Figure 1C).

**Figure 2:**
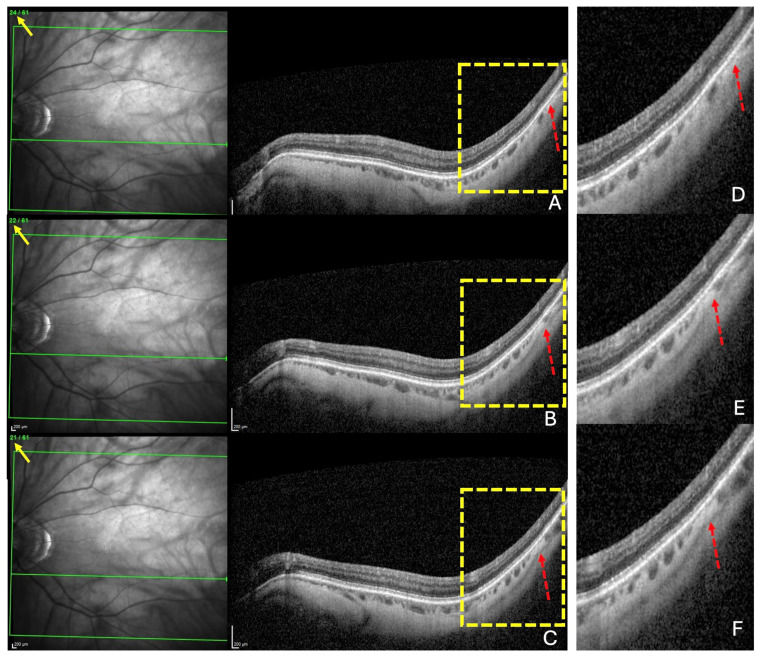
The diagnosis of a choroidal thinning within the band centered by the fovea–Bruch’s membrane opening axis. This diagnosis was retained if a thinning was noted in at least three consecutive sections (dashed red arrows) in (**A**–**C**). Section numbers are highlighted by the yellow arrows in (**A**–**C**). The green lines in (**A–C**) locate the corresponding OCT slices. The yellow rectangles in (**A**–**C**) are presented in (**D**–**F**), respectively.

For each of the three locations, choroidal thinning was considered present when the thinning was noted in at least three adjacent sections (Figure 2A–C), emphasized in (Figure 2D–F).

- Measurement of the choroidal thinning and distance from fovea to thinning.

**Figure 3:**
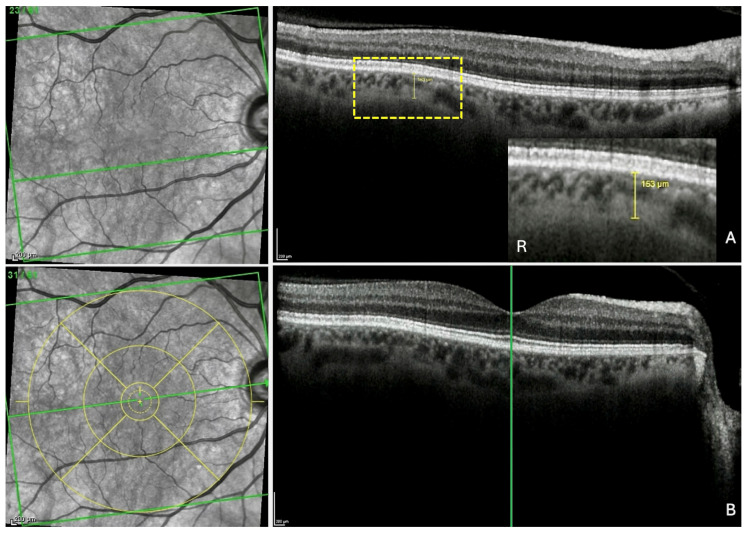
Measurement of the fovea-thinning distance: Step 1—Localization of the fovea in the infrared image. (**A**) After the identification of the choroidal thinning, its mark is preserved in the scan. R: the yellow rectangle is highlighted. (**B**) In the section along the fovea, the vertical reference line is placed on the fovea, which automatically and synchronously displays the horizontal reference line, allowing the localization of the fovea in the infrared image. Finally, the ETDRS grid (yellow circles) is positioned and centered on the fovea in the infrared image to fix its position. The green horizontal line in each infrared image indicates the location of the corresponding slice. The green vertical line in section B locates the fovea which appears in the infrared image as a discontinuity within the horizontal line.

For each of the three locations, the measurement of the thinning detected was taken in the section presenting the greatest thinning, at the level of the thinnest part of this choroidal thinning (Figure 3A). The FT-distance was then measured as follows.

Step 1—Localization of the fovea in the IR image. The ChT thickness measurement was displayed and maintained (yellow line in the OCT section) (Figure 3A). The scan along the fovea was then displayed (Figure 3B). The green vertical reference line was dragged and positioned on the fovea in the OCT scan. This automatically synchronized the precise identification of the fovea in the IR image (green horizontal reference line). Finally, the ETDRS grid (yellow circles) was positioned and centered on the fovea in the IR image, thus fixing its position.

**Figure 4:**
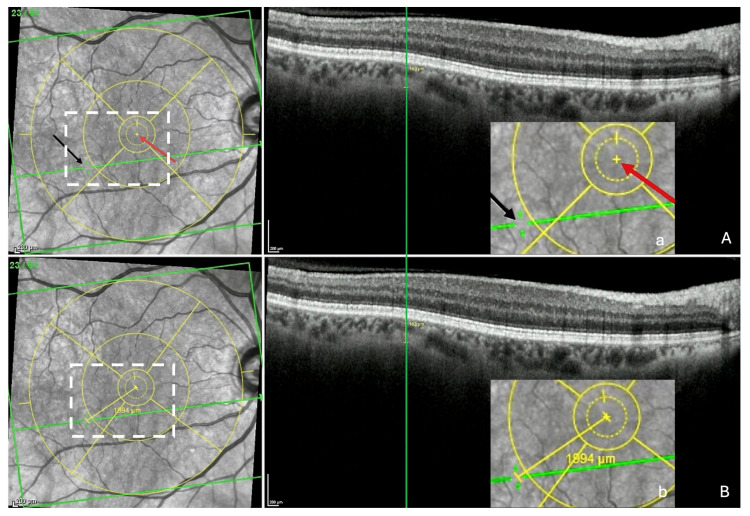
Measurement of the fovea-thinning distance: Step 2—Localization of the thinning in the infrared image and measuring FT-distance. (**A**) The section with the thinning is redisplayed and the green vertical reference line placed at the center of the thinning, which automatically and synchronously displays the green horizontal reference line in the infrared image and reveals a discontinuity (black arrow) within it, corresponding to the location of the thinning. The red arrow shows the location of the fovea. (a = accentuation of the white rectangle displayed in the infrared image). (**B**) Finally, the built-in caliper joins the points between the 2 arrows, automatically displaying the fovea-thinning distance and its value (b = accentuation of the white rectangle displayed in the infrared image). Yellow circles = ETDRS grid.

Step 2—Localization of ChT on the IR image and measurement of the FT-distance. The OCT section with the ChT was displayed again and the green vertical reference line was placed at the center of the ChT (Figure 4A), automatically synchronizing the display of the green horizontal reference line in the IR image and revealing the location of the ChT as a discontinuity (black arrow) within this line. Finally, the built-in caliper was positioned between the mark of the fovea (red arrow) and that of the ChT in the IR image. Automatically, the FT-distance (yellow line) and its value were displayed (Figure 4B).

The position of the thinning relative to the Fo-BMO axis (upper, lower or along this axis) was also noted.

All measurements were taken with the Spectralis^®^ built-in caliper tool.

### 2.2. Analysis Procedure

The OCT from all eyes was analyzed independently by two investigators (Ehongo Adèle (EA) and Jawdat De Togme Georgina (JG)).

Before starting the inclusion, the investigators performed a training session on 20 consecutively selected eyes to refine their diagnostic ability. Once prepared, they proceeded to the agreement analysis using 20 other consecutive eyes, for which a Cohen’s kappa statistic was performed for categorical variables, and Pearson and intraclass correlation coefficients were performed for continuous variables. Finally, they moved to the inclusion phase.

They proceeded until the number of included eyes reached 120. The sample size was not estimated beforehand because this was a pilot study.

The mean value of the two examiners’ measurements was noted for each variable. In the case of disagreement for a variable, the mention “undefined” was noted for this variable.

### 2.3. Statistical Analyses

All the undefined data were excluded from analyses. Descriptive statistics were presented as mean, median, standard deviation or SEM, and range for continuous variables, and as proportions and percentages for discrete variables.

The relationships between the presence of ChT and other variables were assessed. Discrete variables were compared using Fisher’s exact test. The comparison analyses were performed using ANOVA, followed by Tukey post hoc analysis and Pearson’s correlations. The IBM-SPSS V28.0 statistical software was used. A *p*-value lower than 0.05 was considered statistically significant.

## 3. Results

### 3.1. Analysis of Inter-Observer Agreement

The inter-observer analysis performed on 20 eyes showed excellent agreement for the five continuous variables. Pearson correlation coefficients ranged from 0.995 to 0.997 and intraclass correlation coefficients ranged from 0.997 to 1. All were statistically significant (*p* < 0.001) (Table S1).

Additionally, for the categorical variable (the presence of choroidal thinning), there was a strong inter-observer agreement among the raters, evidenced by a Cohen’s Kappa coefficient of 1, also with statistical significance (*p* < 0.001).

### 3.2. Characteristics of the Sample Population

Overall, 120 eyes of 70 subjects were included, 44 (62%) of whom were females. The mean age ± standard deviation was 69.5 ± 9.8 years, range (41–90). The demographic and ocular features of the sample population are summarized in Table 1. A history of phacoemulsification was found in 41 eyes from which refractive data were therefore excluded. No history of refractive surgery was found in the included eyes.

### 3.3. Analysis of Choroidal Thinning at 3 Temporal Sites Relative to the Axis Vertically Aligned with the Fovea

The prevalence of thinning in each of the three temporal choroidal locations is presented in Table 2. For ChT, 112 eyes were defined. It was found in 69.6% (78/112 eyes) of them and was significantly more frequent than the lower or upper thinnings, (*p* < 0.001) in both cases.

### 3.4. Detailed Analysis of Choroidal Thinning at the Vicinity of the Fo-BMO Axis (ChT)

The thickness of the ChT (mean ± SD) was 107.8 ± 6.9 µm, with a range of 9–254 µm.

The proportion of eyes with ChT below the Fo-BMO axis was higher than that along or upper to this axis, but this was not statistically significant, respectively (*p* = 0.093 and 0.133) (Table 3).

No significant difference in ChT thickness was found depending on its position relative to the Fo-BMO axis (Table 3).

The mean CCT was significantly (*p* = 0.018) thinner in eyes with than without a ChT (549.7 ± 37.8 µm versus 569.1 ± 42.2 µm). All other variables (age (*p* = 0.76), gender (*p* = 0.95), and AL (*p* = 0.89)) showed no significant association with ChT.

### 3.5. Analysis of the Distance ChT–Fovea (FT-Distance)

The mean FT-distance value was 3601.9 ± 93.6 µm (mean ± SD), with a range of 1259–5171 µm (Table 4).

The FT-distance was significantly (*p* = 0.003) longer when the ChT was located below rather than above the Fo-BMO axis (3918.9 ± 135.3 µm versus 3201.7 ± 163.5 µm).

**Figure 5:**
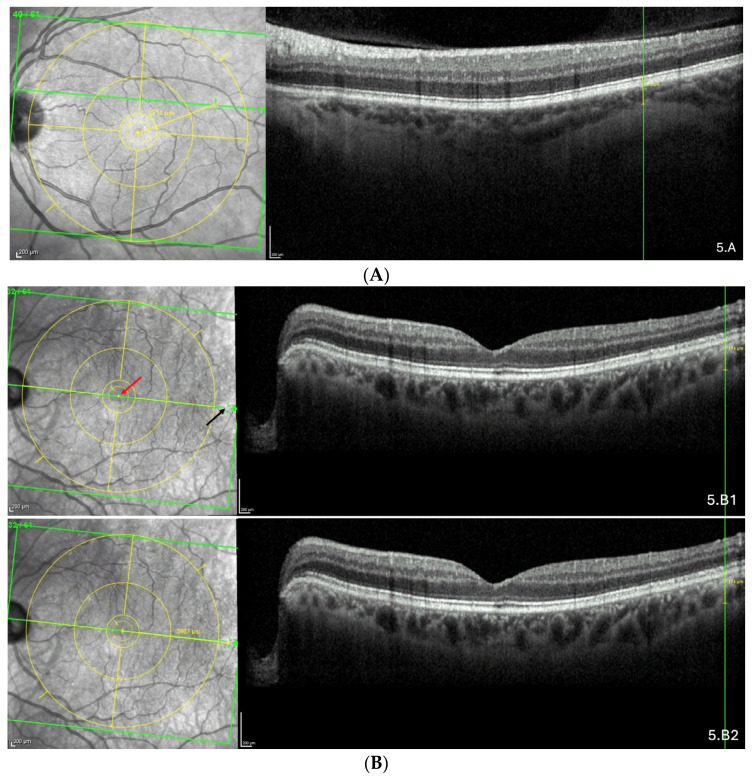

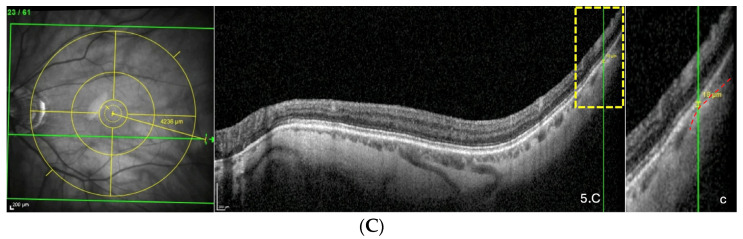
(**A**) Thinning above the FoBMO axis. Thickness of the thinning: 170 µm. Fovea-thinning distance: 2711 µm. Axial length: 22.04 mm. (**B**) Thinning along the FoBMO axis; 5B1. Marks of the fovea (red arrow) and the thinning (black arrow) in the IR image; 5B2. Fovea-thinning distance measured between these marks. Thickness of the thinning: 174 µm. Fovea-thinning distance: 3987 µm. Axial length: 23.94 mm. (**C**) Thinning below the FoBMO axis. Thickness of the thinning: 18 µm. Fovea-thinning distance: 4236 µm. Axial length: 29.86 mm. The yellow box is presented in (**C**). The red curve underlines the posterior choroidal wall. The green horizontal lines in the infrared images locate the OCT sections. (**A**–**C**) The vertical green lines in the OCT sections locate the thinnings which appears as a discontinuity within the horizontal green line of each corresponding infrared image. Abbreviation: FoBMO = Fovea-Bruch’s membrane Opening.

The representative OCT scans of FT-distance in different locations and the corresponding AL and FT-distance values are presented in Figure 5A–C.

**Figure 6:**
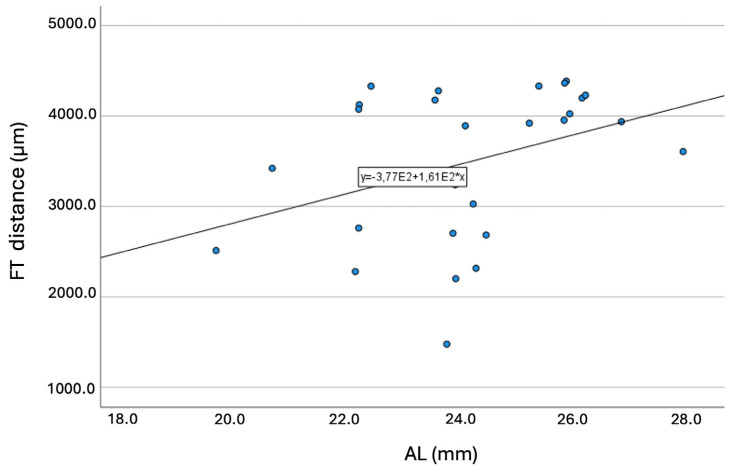
Graph showing the association between the axial length and the fovea-thinning distance. Equation of the regression line: FT-distance (mm) = 377 + 161 × AL. Pearson coefficient = 0.373; *p* < 0.001. Abbreviation: FT = fovea-thinning distance. AL = axial length.

Longer ALs were significantly (*p* = 0.047) associated with longer FT-distances. Figure 6 presents the graph of this association (R = 0.373; *p* < 0.001).

All other variables showed no significant association with FT-distance; (R = 0.097; *p* = 0.402) for CCT, (R = 0.056; *p* = 0.629) for age, and (*p* = 0.555 after *t* test) for gender. Therefore, no adjustments of the FT-distance were performed for these variables.

## 4. Discussion

Predictions suggest that cases of myopia are on the rise worldwide [1]. Its complications have a poor prognosis [3] and explain efforts to slow their progression. This requires a better understanding of their pathogenesis. Unfortunately, many unknowns remain.

Recent ocular biomechanical data have systematically shown that forces acting on the eye wall during eye movements are relevant [7,12] and could contribute to the genesis of certain myopic complications [4,5,7,8,12,13,16].

Particularly, it has been suggested that PS could result from these mechanisms [4,5,13].

In the past, it was assumed that the protrusion of the eyeball characterizing PS resulted from pushing forces directed from inside the eye toward the eye wall [3,17,18,19]. The idea of a traction force acting from the outside has never been put forward and has only been suggested very recently, first for PPS [4,5], then for other types of PS [13].

Based on OCT, a PS is defined by an outward protrusion of the sclera ending in an inward scleral deformation at its edges. In addition, the choroid thins towards the edge of the PS and re-thickens towards the outpouching of the PS [20,21].

The thinning of the choroid due to compressive force applied to the sclera has been previously documented [8,22]. This thinning is followed by a re-thickening at the limits of the compressed zone. It has also been suggested that the choroid thickens where the sclera is pulled outward [4,5,13] because Bruch’s membrane, thanks to its resistance, tends to maintain its plane [23].

**Figure 7:**
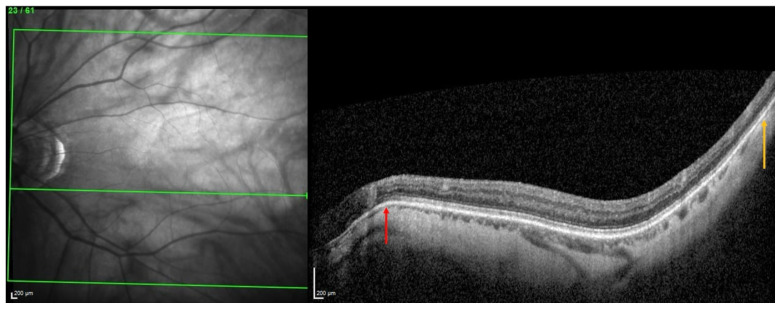
Illustration of two choroidal thinnings related to scleral inward deformations. The choroidal thinning at the peripapillary zone (red arrow) would result from squeezing by the optic nerve sheaths, while the other thinning, temporal to the macular position (yellow arrow), would be related to the action of the inferior oblique muscle.

From this, it was suggested that the underlying pathogenesis of some forms of PS involves inward scleral deformation by compression forces and its outward deformation by traction forces [4,5,13] applied by ON sheaths [4,5] or oblique muscles [13]. The repetition of these deformations over time would lead to a remodeling and fixation of the tissues in the deformed configuration [4,5,13] (Figure 7).

To explore this hypothesis, and before moving on to prospectives studies, we first sought to look for a ChT near and temporal to the macula. Our results revealed a ChT in 70% of eyes, supporting our hypothesis of potential compressive forces at this location in some eyes.

Recently, the thinning of choroidal thickness in the temporal sector has been reported following the instillation of tropicamide + phenylephrine eye drops [15]. This thinning was uniform, unlike the one we present. Additionally, our study subjects did not receive mydriatic eye drops that are not used in our clinic prior to performing Glaucoma Premium Edition measurements.

It is known that choroidal thickness is influenced by several factors, including diabetes [24], uncontrolled hypertension [25], nephritis [26], malignancy [27], or any other history of serious systemic diseases [28,29,30,31,32]. These are general pathologies that would rather induce a generalized choroidal thinning, or at least a uniform thinning of the choroid, since they act globally. Here, we report a sectoral and localized thinning, linked to an inward deformation of the posterior choroidal wall. Prospective studies will allow to accurately exclude the aforementioned factors, the feasibility of which would be limited and biased in the present study due to its retrospective design.

The second unprecedented result came from correlation analyses showing that a thinner CCT was significantly associated with the presence of ChT. As previous results have found a positive correlation between CCT and scleral thickness [33], we suggest that eyes with thinner CCT, and therefore thinner scleral thickness, would also exhibit lower scleral stiffness which would favor local areas of choroidal thinning when the eye wall is subjected to mechanical compression. It is well-known that PS in myopia is favored by reduced scleral rigidity resulting from scleral thinning and remodeling during myopic elongation [17].

We found that the mean age of the participants was 69.8 years. Since the Glaucoma Premium Edition module does not use the Enhanced Depth Imaging (EDI) function [34], its lower acquisition depth does not allow to visualize the posterior choroidal wall of all eyes. This could have contributed to the recruitment of older subjects, as the choroid thins with age [34,35]. Prospective studies using OCT-EDI will help to clarify this point.

Alternatively, this ChT could be an age-related degenerative condition. This could only definitely be established by a longitudinal analysis.

Finally, the influence of age as a single factor could not explain the pathogenesis of this ChT as it is only found in some eyes, suggesting the involvement of other factors.

Our second objective was to measure the FT-distance. This distance was 3601.9 ± 93.6 µm (means ± SD) with a range of 1259 to 5171 µm, meaning that the ChT was positioned 1–5 mm temporal to the fovea, thus coinciding with the insertion distance of the IOM and supporting our hypothesis that this ChT could be promoted by the action of the IOM on the eye wall, in a similar manner to the promotion of PPS via the traction of the ON sheaths [4,5]. As the ChT is only observed in some eyes, additional factors would intervene, such as the force exerted or the resistance of the eye wall to this force, warranting biomechanical studies.

Interestingly, we found a positive correlation between the FT-distance and the AL, indicating that an elongated eyeball would keep thinning away from the fovea, because the insertion of IOM on the eye is fixed while the part of the eye between the ON and this insertion lengthens. This is consistent with previous results showing that the fovea–optic disk distance is positively correlated with AL [36].

Despite the importance of PS as a hallmark of pathologic myopia [37], many unknowns remain; current pathogenetic hypotheses [17,19] do not explain all its characteristics, nor its specific locations on the eye wall which underpin its classification. While we observed ChT in 70% of eyes, PS is described in 0.7% to 2.5% of eyes in population studies [38,39]. Furthermore, PS does not occur in all eyes with high myopia [40]. Finally, some eyes without high myopia develop PS [39,41]. These considerations reveal that PS occurs in eyes with intrinsic susceptibility.

In this study, we discuss one possible promotor of a posterior eye wall deformation, which might be relevant for the pathogenesis of PS. By comparing the distance between the temporal edge of the PS and the fovea with the FT-distance, clues could be gathered regarding their relationship. A correlation between these distances would suggest that the ChT we observed in the present study might be a subtle step of the PS edge feature which would then evolve into an overt PS in predisposed eyes.

Finally, our results showed that the FT-distance was significantly longer when the ChT was located below rather than above the Fo-BMO axis. We suggest that this is related to the path of the IOM along the eye. From its origin at the orbital floor, the IOM runs on the inferior surface of the eye and finally inserts on the posterior inferolateral surface of the eye [14]. This path may present variability, therefore leading to a variability in scleral insertion.

So, just like the perimeter of a curve (the path of the IOM along the eye) from the same starting point (origin of the IOM) for the same length (the length of the IOM), the path with a longer radius of curvature would end (scleral insertion of the IOM) before (below the Fo-BMO axis) the one with the smaller radius of curvature (above the Fo-BMO axis). Additionally, as mentioned above, in the presence of a longer AL, the IOM length is partly depleted by eye elongation, compared to a shorter AL.

It is worth noting that the magnification of retinal images is affected by ocular biometry [42,43], which affects lateral measurements, i.e., those made parallel to the retinal plane in OCT images. Spectralis^®^ OCT, thanks to the built-in HEYEX software (version 6.16.2) based on the Gullstrand schematic eye, automatically accounts for ocular magnification [44,45]. It therefore estimates individual lateral scaling [44,45] according to ocular biometry. However, some authors recommended an additional correction for AL [46], while others have stated that the effect of ocular magnification on scan length would be better accounted for when an individual’s mean keratometry is incorporated into Spectralis^®^ scan acquisition instead of the default setting [45]. Dersch et al. notably acknowledged that a standard method remains to be defined [46]. Furthermore, KIRIK et al. have very recently shown that additional adjustment for AL is not necessary when using Spectralis^®^ OCT [47]. Being a retrospective analysis, our study did not incorporate mean keratometry values, because this should be performed before acquisition [45].

### Limitations and Perspectives

This study is a retrospective investigation and therefore has several inherent limitations. Furthermore, as this is a pilot investigation, and the potential action of the IOM applies to all eyes, we chose to include eyes consecutively, provided that the posterior choroidal wall was visible. We did not specifically analyze the impact of myopia or PS to avoid the risk of bias by using the post-acquisition definition of either entity. Therefore, prospective studies which would clearly specify the definition of each entity (myopia and PS) are warranted.

Interestingly, our results support the hypothesis we formulated and pave the way for potential prospective studies that overcome the limitations of the present study.

First, its retrospective nature led to some missing data. The circadian variation in choroidal thickness that was not considered would not be important because the main outcome (the presence or absence) of localized choroidal wall deformation should not change over the course of the day. Second, the posterior pole module of Spectralis ^®^ that we used would have missed the ChT located outside the acquisition rectangle. Third, as both eyes of some subjects were included, this could interfere with the significance of tests [48]. Fourth, the analysis we performed is extremely simplistic because it neglects other factors that obviously interact. Fifth, the subjects included were from the glaucoma clinic. Therefore, intraocular pressure as a variable has many interferences (treatment or not, one or many drops, …) and has not been considered. Finally, potential other interactions exist and could evolve over time.

The next step consists mainly of a prospective confirmatory study using OCT-EDI to improve the visibility of posterior choroidal wall and to refine the prevalence of the ChT. It would be designed to analyze the impact of the refractive status on ChT and allow the recruitment of younger subjects as well. The procedure would be performed in the same time slot and would take into account all parameters interfering with choroidal thickness.

## 5. Conclusions

This pilot study revealed ChT in 70% of eyes. The CCT was significantly thinner in eyes with than without ChT. The FT-distance was the same value as the IOM insertion distance from the macula. This FT-distance was positively correlated with the AL and was longer in eyes with ChT positioned below the Fo-BMO axis.

These results provide new information regarding a potential link between this posterior choroidal wall deformation and the IOM, which could be relevant in the pathogenesis of some forms of PS, warranting further investigation.

## Figures and Tables

**Table 1:** Demographic and ocular characteristics of the study sample: 120 eyes of 70 subjects.

Parameter		Sample (n)	Mean ± SD	Range
Age (years)		70	69.5 ± 9.8	41–90
Refraction (Diopter)		79	−0.14 ± 2.6	−8.4–5.4
	Myopia (≤−0.5)	30	−2.53 ± 0.4	−8.4–−0.5
	Emmetropia (SE > −0.5 and ≤+0.75)	20	0.11±0.9	−0.4–0.75
	Hyperopia (>+0.75)	29	2.17±0.23	0.88–5.4
Pachymetry (µm)		118	554.7 ± 40.5	452–662
Axial length (mm)		46	23.9 ± 1.6	19.5–27.8

Note: The sample size for each variable corresponds to the number of eyes for which the variable was found in the file. Missing data result from the retrospective design of the study.

**Table 2:** Prevalence of choroidal thinning in the 3 sites of the acquisition rectangle.

Location	Sample Size (n)	Present (n)	Proportion %
Upper part of rectangle	109	25	22.9
Part centered by the Fo-BMO axis	112	78	69.6
Lower part of rectangle	114	29	25.4

Note: The sample size for each variable corresponds to the number of eyes defined for that variable. The proportion of choroidal thinning (Fisher exact test) was significantly different in the zone centered by the Fo-BMO axis than in the superior (*p* < 0.001) or inferior positions (*p* < 0.001). Abbreviation: Fo-BMO = fovea-Bruch’s membrane opening.

**Table 3:** Analysis of ChT around the Fo-BMO axis.

Location	Sample (n)	%	Mean Thickness	SEM	Range
Superior location	23	29.5	130.1	11.6	19.5–248.0
Along the Fo-BMO axis	22	28.2	93.7	12.7	9.0–254.0
Inferior location	33	42.3	101.7	10.7	10.0–248.0
Total	78	100.0	107.8	6.9	9.0–248.0

Note: No significant difference in mean ChT thickness was found depending on its location relative to the Fo-BMO axis (*p* = 0.097 after one-way ANOVA test). Abbreviation: Fo-BMO = fovea-Bruch’s membrane opening.

**Table 4:** Distance between ChT and fovea (FT- distance) according to the Fo-BMO axis.

Location	Sample Size (n)	Mean ± SD	Range
Overall (µm)	78	3601.9 ± 93.6	1259–5171
Superior (µm)	23	3201.7 ± 163.5	1530–4373
Along FoBMO axis (µm)	22	3544.6 ± 165.5	1477–4587
Inferior (µm)	33	3918.9 ± 135.3	1259–5171

Note: The FT-distance was longer below compared to above the Fo-BMO axis (*p* = 0.003). Abbreviation: Fo-BMO = fovea-Bruch’s membrane opening. FT-distance = distance between the fovea and the ChT. ChT = choroidal thinning centered by the fovea-Bruch’s membrane opening axis.

## Data Availability

The data presented in this study are available on request from the corresponding author due to privacy and ethical restrictions.

## Supplementary Materials

The following supporting information can be downloaded at https://www.mdpi.com/article/10.3390/jcm13174978/s1. Table S1: Inter-observer agreement between two readers for 5 continuous variables and *n* = 20 eyes.
