# Supplementary material for: Exploration of Choroidal Thinning Located Temporal to the Fovea: A Pilot Study
Source: J Clin Med. 2024 Aug 23;13(17):4978. doi: 10.3390/jcm13174978 (PMC11396396; doi:10.3390/jcm13174978)
Supplement: Supplementary file 1 [file jcm-13-04978-s001.zip › jcm-3124799-supplementary.pdf]

Journal of
Clinical Medicine

MDPI

Table S1. Inter-observer agreement between two readers for 5 continuous variables and n = 20 eyes.

Parameter	Reader	Mean ± SD (µm)	Intra-class correlation	Range (95% confidence interval)	*p-value*
Superior thickness (n = 20)	R1	210.1 ± 91.9	0.998	0.995 - 0.999	*<0.001*
	R2	207.5 ± 92.7			
Inferior thickness (n = 20)	R1	157.3 ± 77.6	0.998	0.996 - 0.999	*<0.001*
	R2	157 ± 76.7			
Retro-foveal (n = 20)	R1	182.1 ± 77.5	0.997	0.993 – 0.999	*<0.001*
	R2	180.9 ± 74.7			
Thickness of thinning (n = 13)	R1	96.9 ± 49.2	0.998	0.994 – 0.999	*<0.001*
	R2	96. ± 47.4			
Distance fovea-thinning (n = 13)	R1	3465.1 ± 755.8	0.998	0.995 – 1.000	*<0.001*
	R2	3461.2 ± 766.8			

Note: R1 = Reader 1 = AE. R2 = Reader 2 = JG.
